# A Shorter Door-In-Door-Out Time Is Associated with Improved Outcome in Large Vessel Occlusion Stroke

**DOI:** 10.5811/westjem.58946

**Published:** 2023-08-30

**Authors:** Adam Sigal, Derek L. Isenberg, Chadd K. Kraus, Daniel Ackerman, Joseph Herres, Ethan S. Brandler, Alexander Kuc, Jason T. Nomura, Derek R. Cooney, Michael T. Mullen, Huaqing Zhao, Nina T. Gentile

**Affiliations:** * Reading Hospital, Department of Emergency Medicine, West Reading, Pennsylvania; † Lewis Katz School of Medicine at Temple University, Department of Emergency Medicine, Philadelphia, Pennsylvania; ‡ Geisinger Health, Department of Emergency Medicine, Danville, Pennsylvania; § St. Luke’s Health System, Department of Neurology, Bethlehem, Pennsylvania; ∥ Einstein Health System, Department of Emergency Medicine, Philadelphia, Pennsylvania; ¶ State University of New York-Stony Brook, Department of Emergency Medicine, Stony Brook, New York; # Cooper University Healthcare, Department of Emergency Medicine, Camden, New Jersey; ** Christiana Care, Department of Emergency, Newark, Delaware; †† State University of New York-Upstate, Department of Emergency Medicine, Syracuse, New York; ‡‡ Lewis Katz School of Medicine at Temple University, Department of Neurology, Philadelphia, Pennsylvania; §§ Lewis Katz School of Medicine at Temple University, Center for Biostatistics and Epidemiology, Philadelphia, Pennsylvania

## Abstract

**Introduction:**

Endovascular thrombectomy (EVT) significantly improves outcomes in large vessel occlusion stroke (LVOS). When a patient with a LVOS arrives at a hospital that does not perform EVT, emergent transfer to an endovascular stroke center (ESC) is required. Our objective was to determine the association between door-in-door-out time (DIDO) and 90-day outcomes in patients undergoing EVT.

**Methods:**

We conducted an analysis of the Optimizing Prehospital Stroke Systems of Care-Reacting to Changing Paradigms (OPUS-REACH) registry of 2,400 LVOS patients treated at nine ESCs in the United States. We examined the association between DIDO times and 90-day outcomes as measured by the modified Rankin scale.

**Results:**

A total of 435 patients were included in the final analysis. The mean DIDO time for patients with good outcomes was 17 minute shorter than patients with poor outcomes (122 minutes [min] vs 139 min, *P* = 0.04). Absolute DIDO cutoff times of ≤60 min, ≤90 min, or ≤120 min were not associated with improved functional outcomes (46.4 vs 32.3%, *P* = 0.12; 38.6 vs 30.6%, *P* = 0.10; and 36.4 vs 28.9%, *P* = 0.10, respectively). This held true for patients with hyperacute strokes of less than four-hour onset. Lower baseline National Institutes of Health Stroke Scale (NIHSS) score (11.9 vs 18.2, *P* = <.001) and younger age (62.5 vs 74.9 years (*P* < .001) were associated with improved outcomes. On multiple regression analysis, age (odds ratio [OR] 1.71, 95% confidence interval [CI] 1.45–2.02) and baseline NIHSS score (OR 1.67, 95% CI 1.42–1.98) were associated with improved outcomes while DIDO time was not associated with better outcome (OR 1.13, 95% CI 0.99–1.30).

**Conclusion:**

Although the DIDO time was shorter for patients with a good outcome, this was non-significant in multiple regression analysis. Receipt of intravenous thrombolysis and time to EVT were not associated with better outcomes, while male gender, lower age, arrival by private vehicle, and lower NIHSS score portended better outcomes. No absolute DIDO-time cutoff or modifiable factor was associated with improved outcomes for LVOS. This study underscores the need to streamline DIDO times but not to set an artificial DIDO time benchmark to meet.

Population Health Research Capsule What do we already know about this issue?

*Door-in-door-out (DIDO) time (the time spent in the ED at a transferring hospital) is used as a benchmark in stroke patients transferred to a higher level of care.*

What was the research question?

*What is the association between DIDO time and outcomes in patients undergoing endovascular therapy?*

What was the major finding of the study?

*The mean time for patients with good outcomes was 122 minutes vs 139 minutes for patients with poor outcomes (P = 0.04).*

How does this improve population health?

*This study underscores the need to streamline DIDO times but not to set an artificial DIDO-time benchmark for transferring hospitals to meet.*

## INTRODUCTION

Endovascular thrombectomy (EVT) significantly improves outcomes in patients with large vessel occlusion stroke (LVOS).^1^ Time to thrombectomy is a major determinant of neurologic outcome with shorter times associated with better functional outcomes at 90 days.^2^^,^^3^ Key components in managing LVOS include identifying the LVOS, determining eligibility for thrombolytics and EVT, and optimizing management until reperfusion occurs.^4^ Many hospitals in the United States do not have EVT capabilities and must transfer LVOS patients to endovascular stroke centers (ESC) for EVT.^5^

It is not clear how the length of time spent at the transferring non-ESC impacts patient outcomes. Time spent at non-ESC hospitals prior to transfer is defined as the door-in-door-out time (DIDO). Longer DIDO times at the non-ESC may worsen outcomes by delaying recanalization and reperfusion.^6^ However, there is a paucity of evidence on the relationship between DIDO and functional outcomes. In this study, we examined the relationship between DIDO times and 90-day functional outcomes in a multicenter registry. We hypothesized that longer DIDO times would be associated with worse functional outcomes at 90 days.

## METHODS

This study was conducted by the Optimizing Prehospital Stroke Systems of Care-Reacting to Changing Paradigms (OPUS-REACH) consortium. The OPUS-REACH is a consortium of nine health systems in the Northeast US. The structure of the OPUS-REACH consortium has been previously described.^7^ The OPUS-REACH registry includes all patients who underwent EVT at one of the nine ESCs between January 1, 2015–December 31, 2020. An ESC was defined as a thrombectomy-capable stroke center or comprehensive stroke center capable of performing EVT 24 hours a day on an emergency basis. We included all patients who initially presented to a non-ESC and were later transferred to an ESC for EVT. We excluded patients from analysis if their stroke occurred after arrival at the hospital, their 90-day functional outcome was unknown, or if DIDO time at the non-ESC was not available.

We extracted all information from the electronic health records (EHR) at the individual hospitals and submitted to Temple University that serves as the central repository for OPUS-REACH data. Trained research associates or nurses at each of the nine sites used a standardized case report form to abstract data from the EHR. The principal investigator at each of the nine sites verified the data prior to submission to University. The data abstracted were explicit variables without need for interpretation. Missing data was not imputed except for missing 90-day modified Rankin Scale (mRS) scores.

The mRS score was obtained from documentation in the EHR or by estimation by the site investigator using the EHR. If no 90-day mRS score was documented in the EHR or in the hospital’s stroke registry, the site investigator at each ESC reviewed the EHR to identify a clinician note from the 90-day time frame. This could have been a note from neurology, emergency medicine, primary care, physical therapy, or any other licensed clinician. Using this note, the site investigator estimated whether the patient had a good (mRS 0–2) or poor (mRS 3–6) functional outcome. For example, if the EHR reflected that the patient needed care in a skilled nursing faculty this would be coded as a poor outcome while if the note reflected the patient had minimal neurological deficits and walked without assistance, the patient would be assigned a good outcome. This estimation of the mRS score has been previously shown to have good correlation with in-person derived mRS values.^8^ We collected and managed study data using REDCap electronic data capture tools hosted at Temple University.^1^^,^^2^ REDCap (Research Electronic Data Capture) is a secure, web-based software platform designed to support data capture for research studies.

The primary outcome was 90-day functional outcome as assessed by the mRS.^10^^–^^13^ A mRS score of 0–2 was considered a good outcome while a mRS score of 3–6 was considered a poor outcome.

Descriptive summary statistics are presented as means (SD) for continuous variables and as frequencies with percentages for categorical variables. We used the two-sample *t*-test to compare continuous variables and chi-square test to compare categorical variables between good and poor outcomes. Univariate logistic regression was performed to establish potential factors that may contribute to good outcomes. A priori, we defined a *P*-value of <0.05 as significant in the univariate analysis. Multiple variable logistic regression was conducted to determine the association of variables to estimate an odds ratio of a good outcome. We performed statistical analyses with SAS Statistics Software, SAS 9.4 (SAS Institute Inc, Cary, NC). Univariate analysis was performed using *t*-tests to obtain a *P*-value. A priori, we defined *a P-value* of <0.05 as significant. We conducted multiple logistic regression to determine the association of variables to estimate an odds ratio. The institutional review boards of all nine participating institutions approved this study.

## RESULTS

**Figure 1.**
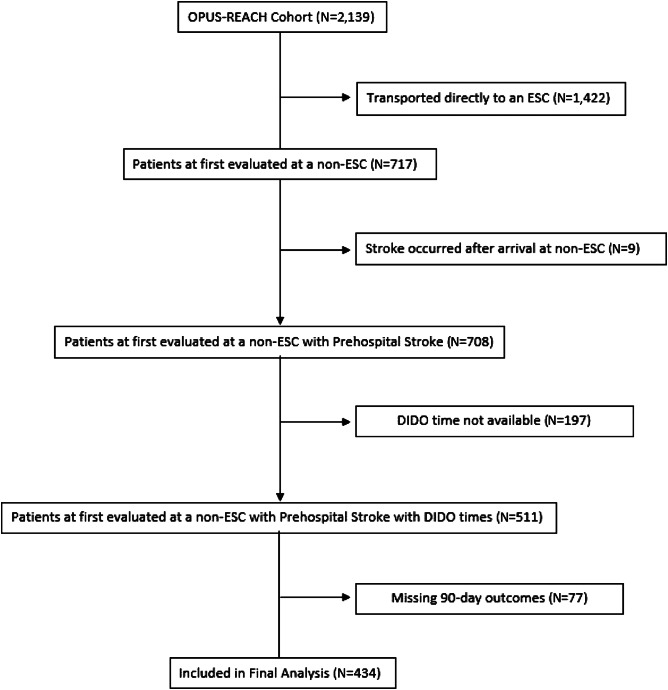
Enrollment diagram. Of the initial cohort of 2,129 patients, 434 were included in the final analysis based on complete DIDO times and neurologic outcomes. ESC, endovascular stroke center.

Of the 2,139 patients in the OPUS-REACH registry, we included 434 in the final analysis (Figure 1). Patients were predominantly White with an even distribution by gender. The median age was 71 years (Table 1). Of the 378 patients for whom the time of vascular imaging was known, 56% of patients had vascular imaging performed at the transferring hospital.

**Table 1.** Demographics.

Demographic	N = 434 (%)
Race	
American Indian/Alaska Native	1 (0.2)
Black or African American	62 (15.0)
White	347 (83.8)
Asian/Native Hawaiian/Pacific Islander	2 (0.5)
Other	2 (0.5)
Unknown	22 (5.0)
Hispanic ethnicity	
Yes	16 (3.7)
No	416 (96.3)
Unknown	4 (0.9)
Age	
Mean (SD)	70.7 (16.2)
Range	25–103
Gender	
Male	211 (48.4)
Female	225 (51.6)
Baseline Patient Characteristics	
Mean initial NIHSS (SD)	16.1 (8.1)
Mean time from last known well to arrival at non-ESC in minutes (SD)	276 (325)
Mean time from last known well to EVT in minutes (SD)	505 (344)

*NIHSS*, National Institutes of Health Stroke Scale; *EVT,* endovascular thrombectomy.

We found that 33% of patients had a good outcome (mRS 0–2). Patients who had favorable 90-day outcomes had a lower baseline NIHSS score than those with poor outcomes (NIHSS 11.9 vs 18.2, *P* < 0001). Patients with better outcomes were more likely to arrive by private transport than by emergency medical services (EMS) (56.5 vs 30.6%, *P* = 0.004). Overall, patients with good outcomes had a 17-minute faster DIDO time than patients with poor outcomes (122.6 minutes [min] vs 139.8 min, *P* = 0.037). This trend, however, did not meet statistical significance when analyzed with DIDO cutoff times of ≤60, ≤90, or ≤120 min (Table 2).

**Table 2.** Univariate analysis of entire cohort.

Variable	Overall (N = 434)	Good (N = 144)	Poor (N = 290)	*P*-value
	434	144	290	
Mean DIDO time at non-ESC (SD)	**134.1 (86.7)**	**122.6 (74.0)**	**139.8 (91.9)**	**0.04**
Means of arrival to non-ESC (n = 415)				**<.001**
EMS	369 (88.9)	113 (30.6)	256 (69.4)	
Private Vehicle	46 (11.1)	26 (56.5)	20 (43.5)	
Received IVT				0.08
No	263 (60.6)	79 (30.0)	184 (70.0)	
Yes	171 (39.4)	79 (38.0)	106 (62.0)	
Vascular imaging performed at non-ESC (n = 324)				0.73
No	141 (43.5)	46 (32.6)	95 (67.4)	
Yes	183 (56.5)	63 (34.4)	120 (65.6)	
Level of non-ESC (n = 434)				0.90
Not certified	59 (13.6)	20 (33.9)	39 (66.1)	
PSC	375 (86.4)	124 (33.1)	251 (66.9)	
Race (n = 412)				0.55
American Indian	1 (0.2)	0	1 (100.0)	
Asian/Pacific Islander	2 (0.5)	0	1 (100.0)	
Black	62 (15.1)	23 (37.1)	39 (62.9)	
White	345 (83.7)	111 (32.2)	234 (67.8)	
Other	2 (0.5)	0	2 (100.0)	
Hispanic ethnicity (n = 430)				0.57
No	415 (96.5)	137 (33.0)	278 (67.0)	
Yes	15 (3.5)	6 (40.0)	9 (60.0)	
Mean age (SD)	70.8 (15.1)	62.5 (15.0)	74.9 (13.5)	**<.001**
Gender				**0.02**
Female	224 (51.5)	63 (28.1)	161 (71.9)	
Male	211 (48.5)	81 (38.4)	130 (61.6)	
DIDO time at non-ESC				
≤60 minutes (min)	28 (6.5)	13 (46.4)	15 (53.6)	0.12
>60 min	406 (93.6)	131 (32.3)	275 (67.7)	
≤90 min	140 (32.3)	54 (38.6)	86 (61.4)	0.10
>90 min	294 (67.7)	90 (30.6)	204 (69.4)	
≤120 min	247 (56.9)	90 (36.4)	157 (63.6)	0.10
>120 min	187 (43.1)	54 (28.9)	133 (71.1)	
LKW to arrival at non-ESC (n = 401)				0.31
Mean time in minutes (SD)	278.83 (330.1)	254.63 (300.1)	290.57 (343.6)	
LKW to arrival at non-ESC in <4 hours (n = 266)		88 (33.1)	178 (66.9)	0.80
LKW to EVT (n = 394)				0.28
Mean time in minutes (SD)	497.4 (334.8)	471.4 (307.5)	510.4 (347.5)	
Initial NIHSS score (n = 430) [SD]	16.1 (8.1)	11.9 (7.3)	18.2 (7.7)	**<.001**

*DIDO*, door-in-door-out; *ESC*, endovascular stroke center; *EMS*, emergency medical services; *IVT*, intravenous thrombolysis; *PSC*, primary stroke center; *LKW*, last known well; *EVT*, endovascular thrombectomy; *NIHSS* National Institutes of Health Stroke Scale.

We performed a subgroup analysis for patients who arrived at the non-ESC within four hours of the onset of their strokes (hyperacute stroke). Here, we saw no difference in the mean DIDO times between the group with good outcomes and the group with poor outcomes (Table 3). Again, lower age and lower initial NIHSS score portended better outcomes. On multiple variable analysis, we found that age and lower baseline NIHSS score were the only variables associated with improved outcomes in LVOS patients. There were no modifiable factors associated with improved outcomes. (Table 4).

**Table 3.** Univariate analysis of patients with hyperacute stroke presentation (onset to arrival of non-endovascular stroke center of <240 minutes).

Variable	Total	Good outcome	Poor outcome	*P*-value
	265	87	178	
Mean DIDO time (SD)	126.3(71.2)	116.6 (62.4)	131.1 (74.9)	0.12
Means of arrival to Nnon-ESC (n = 254)				
EMS	227 (89.4)	67 (29.5)	160 (70.5)	**<.001**
Private vehicle	27 (10.6	15 (55.6)	12 (44.4)	
Received IVT				
No	106 (40.0)	28 (26.4)	78 (73.6)	0.07
Yes	159 (60.0)	59 (37.1)	100 (62.9)	
Vascular imaging performed at non-ESC (n = 200)				
No	88 (44.0)	28 (31.8)	60 (68.2)	0.82
Yes	112 (56.0)	34 (30.4)	78 (69.6)	
Level of non-ESC	265			
Not certified	41 (15.5)	13 (31.7)	28 (68.3)	0.87
PSC	224 (84.5)	74 (33.0)	150 (67.0)	
Race (n = 249)	249			
American Indian	1 (0.4)	0	1 (100)	0.83
Asian or Pacific Islander	1 (0.4)	0	1 (100)	
Black	32 (12.9)	11 (34.4)	21 (65.6)	
White	214 (85.9)	68 (31.8)	146 (68.2)	
Other	1 (0.4)	0	2 (100)	
Hispanic ethnicity (n = 263)				
No	252 (95.8)	84 (33.3)	168 (66.7)	0.68
Yes	11 (4.2)	3 (27.3)	8 (72.7)	
Mean age				**<.001**
Mean (SD)	70.87 (15.14)	62.86 (14.83)	74.78 (13.71)	
Gender (n = 245)				**0.04**
Female	146 (55.1)	40 (27.4)	106 (72.6)	
Male	119 (44.9)	47 (39.5)	72 (60.5)	
DIDO time at non-ESC				
≤60 minutes (min)	16 (6.0)	6 (37.5)	10 (62.5)	0.68
>60 min	406 (93.6)	131 (32.3)	275 (67.7)	
≤90 min	91 (34.3)	34 (37.4)	57 (62.6)	0.26
>90 min	294 (67.7)	90 (30.6)	204 (69.4)	
≤120 min	158 (59.6)	57 (36.1)	101 (63.9)	0.17
>120 min	187 (43.1)	54 (28.9)	133 (71.1)	
LKW to arrival at non-ESC				0.35
Mean (SD)	79.31 (53.2)	74.95 (53.4)	81.43 (53.1)	
LKW to EVT (n = 260)				0.97
Mean (SD)	301.13 (114.2)	300.69 (114.9)	301.35 (114.1)	
Initial mean NIHSS score (SD) [n = 263]	263			

*DIDO*, door-in-door-out; *ESC*, endovascular stroke center; *IVT*, intravenous thrombolysis; *EMS*, emergency medical services; *PSC*, primary stroke center; *LKW*, last known well; *EVT*, endovascular thrombectomy.

**Table 4.** Multiple regression analysis for entire cohort.

	Odds ratio	95% Confidence limits	
DIDO	1.13	0.99	1.30
Not in same hospital system	1.98	1.18	3.31
NIHSS	1.67	1.42	1.98
Age	1.71	1.45	2.02

Hyperacute Stroke Presentation (onset to arrival of non-ESC of <240 minutes)			
	Odds ratio	95% Confidence limits	
DIDO	1.22	0.99	1.50
Not in same hospital system	3.02	1.49	6.11
NIHSS score	1.76	1.41	2.20
Age	1.80	1.43	2.25

*DIDO*, door-in-door-out; *NIHSS*, National Institutes of Health Stroke Scale.

## DISCUSSION

Although patients with good outcome had slightly faster DIDO times overall, we could find no specific DIDO threshold up to 120 min that was associated with improved outcomes at 90 days whether early or late in the stroke process. The initial NIHSS score and the patient’s age were more predictive than DIDO time for favorable outcome. We found some surprising factors that were not associated with outcomes among our patients. Arrival by EMS was significantly associated with improved outcomes in our cohort. Arrival by EMS was closely linked to a higher NIHSS score. Therefore, EMS arrival was not significant on multiple variable analysis. Receipt of intravenous thrombolysis (IVT) was borderline significant on univariate analysis. This may be related to a small sample size or to the fact that the mean arrival time to the non-ESC was greater than 4.5 hours, making many patients ineligible for IVT.

Most interesting, time to EVT was not associated with better outcomes, despite prior research showing delays to EVT were associated with worse outcomes. For example, Jahan et al found that each 15-min delay to EVT was associated with worse outcomes.^14^ However, this effect only persisted up to 270 min. Therefore, the longer time to EVT in our cohort may have diluted effects of time to EVT. Although DIDO time is linked to EVT time, there are many more time intervals involved in stroke onset to EVT. It may be that time from hospital arrival to EVT or time from imaging to EVT are more important factors in outcomes after LVOS.

The model for transferring patients to higher levels of care and concern that delays may impact long-term function is an adaptation of the models developed for managing acute ST-elevation myocardial infarction (STEMI). Among STEMI transfer patients, those with prolonged DIDO times had increased mortality at 30 days.^15^ Transferred patients with DIDO times <30 min had both shorter perfusion times and lower in-hospital mortality, with the odds ratio of 1.56 for those with times >30 min.^16^

In one metanalysis of nearly 16,000 patients, the authors found that those with DIDO time of <30 min had a 2.8% 30-day mortality compared to 6.0% for patients with DIDO times of >30 min.^17^ However, DIDO times for transferred patients undergoing primary percutaneous intervention rarely meet the recommended 30-min goal.^18^^,^^19^ Common delays in transfer include transport delays, diagnostic uncertainty, and clinical instability prior to transfer.^20^

As regional stroke systems of care develop, DIDO has been proposed as a metric for transferring hospitals to follow. Choi et al demonstrated that a primary stroke center can achieve median DIDO times of <60 min.^21^ However, compared to the STEMI transfer process, the diagnosis of LVOS is more complex. Whereas a STEMI diagnosis can be made based on a 12-lead electrocardiogram and an abbreviated history, the diagnosis of an LVOS requires a thorough neurologic exam, advanced computed tomography, radiologic interpretation, and consultation with a neurologist. Currently the American Heart Association (AHA) makes no recommendations as to an optimal DIDO time for LVOS.^1^ While the benefit of EVT is time dependent early on in LVOS, and systems of care should prioritize rapid transfer of LVOS patients to an ESC, more research is needed to identify a clinically meaningful DIDO threshold, prior to adopting national benchmarks. From our data, we cannot recommend a specific DIDO time for LVOS patients in an analogous manner to STEMI patients.

## LIMITATIONS

Our study has several limitations. As it was a retrospective study, we did not have complete data on all the patients transferred to thrombectomy centers for EVT. We were missing DIDO times for 197 patients largely because of missing data from the transferring hospitals. As data was collected retrospectively, records from the transferring hospital were sometimes missing from the EHR of the ESC. In addition, the three-month outcome was based on a mRS score that at times was estimate-based. Prior studies have validated this methodology, however.^8^

In addition, we included patients cared for by nine health systems and over 100 non-ESCs. Although most followed standard AHA/American College of Cardiology stroke guidelines for care and are designated as ESCs, there are variations in prehospital EMS communications, transfer processes, initial imaging, and thrombolysis process at the initial hospitals from which they receive patients. However, the inclusion of many health systems in a wide contiguous geographic region improves generalizability and provides information about outcomes in routine clinical practice, rather than clinical trials. All patients in our study were known to have LVOS and underwent EVT. We do not know the DIDO times for patients who were transferred but did not undergo EVT.

## CONCLUSION

Although the mean door-in-door-out time is shorter for patients with good functional outcomes after large vessel occlusion stroke there is no absolute DIDO cutoff time up to 120 minutes that improved 90-day functional outcome for patients with an LVOS undergoing endovascular thrombectomy. Before establishing DIDO metrics for referring hospitals, more research is needed on factors that improve clinical outcomes. The most important predictor of 90-day outcomes is the patient’s age and initial NIHSS score. Non-endovascular stroke centers should focus on following AHA/American Stroke Association guidelines for initial stroke care, including intravenous thrombolysis administration, and appropriate transfer to an ESC.
